# Comparison of the siRNA and mRNA Carrying Capacity of Quaternary Ammonium β-Cyclodextrin Polymer and Polyethylenimine

**DOI:** 10.3390/pharmaceutics18060713

**Published:** 2026-06-10

**Authors:** Ágnes Rusznyák, Péter Magyar, Virág Dajka, Alexandra Gyöngyösi, István Lekli, György Vámosi, Milo Malanga, Éva Fenyvesi, Lajos Szente, Judit Váradi, Ildikó Bácskay, Eszter Puhl, Ferenc Fenyvesi

**Affiliations:** 1 Department of Molecular and Nanopharmaceutics, University of Debrecen, Rex Ferenc St. 1, H4002 Debrecen, Hungary; pmagyar20@gmail.com (P.M.); dajvirag@gmail.com (V.D.); fenyvesi.ferenc@pharm.unideb.hu (F.F.); 2 Department of Pharmacology, University of Debrecen, Rex Ferenc St. 1, H4002 Debrecen, Hungary; gyongyosi.alexandra@pharm.unideb.hu (A.G.); lekli.istvan@pharm.unideb.hu (I.L.); 3 Department of Biophysics and Cell Biology, University of Debrecen, Egyetem Square 1, H4032 Debrecen, Hungary; vamosig@med.unideb.hu; 4 CycloLab Ltd., Illatos St. 7, H1097 Budapest, Hungary; milo.malanga@carbohyde.com (M.M.); fenyvesi.e@cyclolab.hu (É.F.); szente@cyclolab.hu (L.S.); 5 Department of Pharmaceutical Technology, University of Debrecen, Rex Ferenc St. 1, H4002 Debrecen, Hungary; varadi.judit@pharm.unideb.hu (J.V.); bacskay.ildiko@pharm.unideb.hu (I.B.); puhl.eszter@phd.semmelweis.hu (E.P.); 6 Institute of Healthcare Industry, University of Debrecen, Rex Ferenc St. 1, H4002 Debrecen, Hungary

**Keywords:** siRNA, mRNA, cyclodextrin, polymer, gene delivery

## Abstract

**Background/Objectives**: Intracellular delivery of RNA molecules is challenging. To solve this problem, many carrier systems are available, which are based on liposomes or polymers. Cyclodextrins are widely used excipients to increase the solubility of small molecules, but their polymer derivatives are able to deliver macromolecules. In the present study, we aimed to investigate and compare the siRNA and mRNA carrying capacity of a cationic quaternary ammonium β-cyclodextrin polymer (QABCDPS) and polyethylenimine (PEI). **Methods**: Cytotoxicity of the polymers was tested by the MTT method. Polyplexes were formulated with different nitrogen/phosphate ratios (NP), and their physicochemical properties were examined using dynamic light scattering and zeta potential measurements. Cellular internalization and intracellular effects of the polyplexes were investigated by confocal microscopy and flow cytometry. **Results**: QABCDPS exhibited lower toxicity compared to PEI, effectively binding both siRNA and mRNA and delivering them into vesicles in the cytoplasm, but showing different internalization patterns. Polyplexes formed with PEI showed stronger biological effect than those with QABCDPS, which can be attributed to the strength of interactions facilitated by the polymers. **Conclusions**: In summary, QABCDPS is a low-toxicity carrier that shows some promise for mRNA delivery but is ineffective for siRNA silencing under the tested conditions and requires further structural optimization.

## 1. Introduction

Recently, RNA-based therapies have become crucial in treating a wide range of diseases. Small interfering RNA (siRNA) and messenger RNA (mRNA) have attracted the most attention. siRNA is a double-stranded, non-coding RNA molecule, which consists of 21–25 base pairs. It plays a role in gene silencing, by specifically interfering with the target mRNA causing its degradation, therefore inhibiting the translation [1]. In contrast, mRNA is a single-stranded RNA molecule, consisting of several hundred or thousand base pairs, which encodes for the corresponding protein [2]. With the appearance of the mRNA vaccines against the SARS-CoV-2 virus, the research into oligonucleotide-based therapies has expanded greatly. While RNA-based therapies have demonstrated significant promise, the stability and the transfection of these molecules present many obstacles. Due to the large molecular size and polyanionic nature of the naked RNA, it is difficult to deliver into the cells and it may be unstable and immunogenic in the blood stream [3]. To overcome these problems and for successful therapy, one of the potential solutions is an efficient and safe carrier system. These systems must protect the RNA from nucleases and evade the immune system [4]. Several delivery systems are available or under development. Most of them are lipid-based carrier systems (lipoplexes), but they may present many disadvantages, such as nausea, weakness, allergic reactions, anaphylaxis or thrombosis [5]. To minimize these drawbacks, polymer-based delivery systems (polyplexes) can be used, involving cationic polymers [6]. While both siRNA and mRNA delivery face common challenges and barriers, including enzymatic degradation, limited cellular uptake, and endosomal entrapment, their intracellular requirements differ substantially. siRNA must be efficiently released into the cytoplasm and incorporated into the RNA-induced silencing complex (RISC) to mediate gene knockdown, with additional considerations such as strand selection and off-target effects [7]. In contrast, mRNA delivery requires not only cytoplasmic release but also efficient translation by ribosomes, making it more sensitive to molecular size, structural stability, and innate immune activation [8]. One of the major challenges in RNA delivery is the development of an optimal delivery system that ensures efficient cellular uptake while facilitating endosomal escape [9]. Endosomal escape of RNAs and RNA carriers remains a major unresolved challenge. According to previous studies, only 1–2% of siRNA escapes into the cytosol from lipid nanoparticles after endosomal uptake [10,11].

Cyclodextrin monomers are widely used excipients to increase solubility and bioavailability of small lipophilic guest molecules [12], but cyclodextrin polymers are even able to carry macromolecules [13]. Previously, we showed that both cyclodextrin monomers and a soluble β-cyclodextrin polymer can enter the Caco-2 intestinal epithelial and HeLa cervical cells via fluid-phase endocytosis. These derivatives did not induce the NF-κB inflammatory pathway or autophagy at non-toxic concentrations, and can be found in the lysosomes [14,15,16,17]. Cyclodextrin-based delivery systems were designed for RNA delivery; however, cyclodextrin polymers with different structures were not fully characterized for this purpose. Application of cyclodextrins in RNA delivery systems has beneficial effects, such as the stabilization of siRNA by cationic cyclodextrins via electrostatic interactions [18] and the reduction in toxicity through the incorporation of cyclodextrins [19]. Among polymerized cyclodextrins, linear cationic β-cyclodextrin-containing polymers were used earlier as a delivery vehicle of plasmid DNA [20]. Monfared et al. showed that the cationic hyperbranched cyclodextrin-based polymer has no cytotoxic effects and can efficiently deliver mRNA to melanoma cancer cells [21]. Another approach for the delivery of RNA by cyclodextrin-based delivery systems is the application of amphiphilic cyclodextrin derivatives. These molecules, also known as ionizable cyclodextrins, have self-assembly properties, which means that these molecules have the ability to form nanosized carriers spontaneously without surfactants. These nanoparticles can improve the drug release profile, the cellular uptake, and the tumoral penetration [22,23]. O’Driscoll et al. showed that amphiphilic cationic β-cyclodextrin systems demonstrated a significant antitumor efficacy against prostate cancer [24] and against acute myeloid leukaemia [25].

The simple formation of polyplexes with cyclodextrin polymers makes them a promising tool for RNA delivery. Additionally, there is an intensive search for a suitable polymer that is characterized for its siRNA and mRNA delivery capabilities. In the present study, our goal was to test the siRNA and mRNA carrying properties of a soluble, cross-linked quaternary ammonium beta-cyclodextrin polymer (QABCDPS). This polymer has permanent positive charges due to its trimethylamino groups. Recently, this type of cyclodextrin polymer and a β-cyclodextrin polymer functionalized with primary amine groups (PA-polymer) were characterized for their siRNA delivery capabilities [26]. PA-polymer showed enhanced performance on siRNA delivery into cells; however, their mRNA delivery capacity was not tested. Nazli et al. also investigated the siRNA binding efficacy of a poly(glycidyl methacrylate)modified mono(6(diethylenetriamine)-6-deoxy)-β-CD [27]. This derivative also demonstrated enhanced binding, biocompatibility, and effective gene silencing, but the mRNA binding capacity of this was not examined.

In our study, 25 kDa polyethylenimine (PEI) was utilized as a control polymer. This type of PEI is considered as the “gold standard” in polyplex formulations and used extensively to deliver nucleic acids [19]. The branched form showed the most effective gene delivery and DNA condensation [28]. The mechanism of liberation of nucleic acids after cellular uptake of PEI polyplexes is explained by the proton sponge effect, which is in the background of PEI’s intrinsic endosomolytic activity. Unfortunately, the greatest disadvantage of PEI is its cytotoxic effect, due to the swelling and rupture of the endosomal membrane and cell membrane permeabilization. We evaluated the cytotoxicity of both PEI and QABCDPS, characterized the formulated polyplexes, examined their cellular uptake, and assessed the intracellular effects by measuring the expression of glyceraldehyde 3-phosphate dehydrogenase (GAPDH) and green fluorescent protein (GFP). Major differences were found in their behavior and effects, which can be explained by their chemical structures. The novelty of this study lies in the systematic investigation and comparison of a commercially available, low-cytotoxic, cationic cyclodextrin polymer with respect to its siRNA and mRNA complexation and delivery capabilities, with the aim of evaluating its potential as a novel nucleic acid delivery platform. The cytotoxicity, cellular uptake, effect on endosomal escape, and siRNA and mRNA delivery properties of QABCDPS were characterized and compared to PEI.

## 2. Materials and Methods

### 2.1. Materials

Quaternary ammonium β-cyclodextrin soluble polymer crosslinked with epichlorohydrin (QABCDPS) was synthesized at CycloLab Ltd. (Budapest, Hungary) according to the following protocol. Sodium hydroxide and dry BCD (~10:1 2:1 molar ratio) were dissolved in deionized water. After obtaining a clear yellow solution, the reaction mixture was heated to 65 °C. Epichlorohydrin (~10:1 molar ratio related to BCD) was added over 2 h, and then the reaction mixture was stirred for an additional 2 h, maintaining this temperature. The reaction mixture was cooled to room temperature, and then further sodium hydroxide was added. After obtaining a clear yellow solution, the reaction mixture was cooled down to 10 °C. Glycidyltrimethylammonium chloride (GTAC, ~4:1 molar ratio related to BCD) was added during an hour, maintaining this temperature. After the addition, the reaction mixture was stirred at room temperature for two days. The reaction mixture was heated for 3 h (to 70 °C) to decompose the remaining GTAC and was neutralized with HCl solution (pH was set to 6). After the mixture was subjected to dialysis (molecular weight cut off: 14.000), the solid product was obtained by lyophilization. The polymer has the following properties: MW~40.000 (measured by Malver light scattering equipment, Malvern Panalytical, Malvern, UK); CD content: 70–75% (measured by NMR) degree of substitution (DS) 1.5–2.0/CD unit (measured by capillary electrophoresis), the QA moieties can be located on the CD ring and on the crosslinking bridges, and on the dihydroxypropyl side chains. The fluorescein-labeled derivative of QABCDPS was the product of CycloLab Ltd. (Budapest, Hungary). The Silencer^®^ siRNA Labeling Kit, Lipofectamine™ RNAiMAX Transfection Reagent, Lipofectamine™ MessengerMAX™ Transfection Reagent, and Opti-MEM™ I Reduced Serum Medium were obtained from Thermo Fisher Scientific (Waltham, MA, USA). Polyethylenimine (PEI; branched, MW~25.000) and Predesigned human GAPDH siRNA (Sequence Start: 230) were the products of Sigma-Aldrich Ltd. (Budapest, Hungary) and CleanCap EGFP mRNA was the product of Tebubio (Le Perray-en-Yvelines, France). All other materials were the products of Sigma-Aldrich Ltd. (Budapest, Hungary).

### 2.2. Cell Cultures

The two applied cell lines (Caco-2 intestinal epithelial and HeLa human cervix epithelioid carcinoma cells) were obtained from the European Collection of Cell Cultures (ECACC, Salisbury, UK). The cells were cultured at 37 °C in a humidified incubator with 5% CO_2_. Dulbecco’s Modified Eagle’s Medium (DMEM; Sigma-Aldrich Ltd., Budapest, Hungary) was used as the growth medium, supplemented with 10% heat-inactivated fetal bovine serum, 1% non-essential amino acids, and 1% penicillin–streptomycin solution (all from Sigma-Aldrich Ltd., Budapest, Hungary). The passage numbers ranged from 30 to 45 for Caco-2 cells and from 15 to 50 for HeLa cells.

### 2.3. Cytotoxicity of the Polymers

The cytotoxic effects of the applied polymers were evaulated by MTT assay. HeLa cells were seeded into 96-wells plates (VWR International Inc., Debrecen, Hungary) at a density of 5 × 10^3^ cells/well. After the cells reached the appropriate confluence, the medium was removed, and the cells were treated with the polymer solution in different concentrations (4, 20, 40, 200, 400, and 4000 µg/mL QABCDPS, and 2.5, 12.5, 25, 125, 250, and 1250 µg/mL PEI) for 48 h at 37 °C in Opti-MEM media. Opti-MEM media was used as a negative and 1% Triton X-100 solution as a positive control. Following the treatment period, the test solution was discarded and a 0.5 mg/mL MTT solution was added to each well. The plate was incubated for an additional 3 h at 37 °C. After incubation, the MTT solution was completely removed and 0.1 mL of an isopropanol:hydrochloride acid 1 M (25:1) solution was added to each well to lyse the cells and dissolve the formazan crystals for absorbance measurements at both 570 and 690 nm using a Thermo-Fisher Multiskan Go (Thermo-Fisher Scientific, Waltham, MA, USA) microplate reader. Cell viability was expressed as a percent of the cell viability of the untreated control cells. All experiments were performed independently in triplicate, each with six technical replicates.

### 2.4. Polyplex Formulation and Characterization

#### 2.4.1. Polyplex Formulation

Polymer/siRNA and polymer/mRNA polyplexes were prepared by mixing QABCDPS (MW~40.000) or PEI (MW~25.000) and GAPDH siRNA (MW~13.000) or mRNA (MW~319.000) at different nitrogen/phosphate (NP) ratios, in RNase- free water. The applied range was determined in preliminary experiments based on the estimated amounts of amino and phosphate groups in the molecules. The complexes were incubated for 20 min at room temperature and were then diluted to the appropriate concentration.

#### 2.4.2. Dynamic Light Scattering (DLS) and Zeta Potential

Dynamic light scattering and zeta potential measurements were carried out by ZetaSizer Nano ZS (Malvern Instruments Ltd., Malvern, UK) in backscattering mode. All measurements were performed in high concentration cell cuvettes, at a 173° scattering angle with the temperature controlled at 25 °C. Three consecutive measurements were carried out for each sample. The siRNA concentration was 100 µM, and the mRNA concentration was 1 µg/mL. The polymer solutions were added in increasing amounts to the RNA solutions to form polyplexes at different NP.

#### 2.4.3. Gel Electrophoresis

The interaction between the RNA and the polymers was investigated by gel electrophoresis. The agarose gel was prepared in Tris base, acetic acid and ethylenediaminetetraacetic acid (TAE buffer) by dissolving 1 g of agarose in 100 mL buffer containing 2 µL GelRed ^®^ 10,000× (Biotium, Fremont, CA, USA). Polyplexes were prepared at different NP, keeping the concentration of siRNA at 100 nM and mRNA at 0.10 µg/µL. A 20 µL sample was mixed with 5 µL gel loading buffer and loaded into the wells of the gel. Electrophoresis was performed at 80 V for 80 min and results were visualized under UV with ChemiDoc Imaging Systems (BioRad, Hercules, CA, USA).

### 2.5. Investigation of the Cellular Uptake

In these experiments, fluorescently labeled GAPDH siRNA was used. The siRNA molecule was labeled with Cy3 dye, according to the Silencer^®^ siRNA Labeling Kit specification.

#### 2.5.1. Confocal Microscopy

For the examination of cellular uptake, 80,000 Caco-2 cells/well were seeded on round glass cover-slips placed into 24 well plates. Once reaching the desired confluence, the cells were treated for 30 min, 4 h, or 24 h at 37 °C with polyplex solutions containing 5 µM Cy3 labeled GAPDH siRNA. After the incubation period, the cells were fixed with 3.7% paraformaldehyde solution for 15 min at room temperature and cell nuclei were stained with 283 nM DAPI for 10 min at room temperature. As a final step, the round glass cover-slips were glued to glass microscope slides. Between every step, cells were washed four times with Hank’s Balanced Salt Solution (HBSS). Confocal microscopy measurements and analyses were carried out on a Zeiss LSM880–Airy-scan confocal laser scanning microscope (Carl Zeiss Microscopy GmbH, Jena, Germany). To eliminate spectral crosstalk, the samples were illuminated with three different excitations in “multi-track” mode in an alternating fashion (UV: 351.1 nm and 363.8 nm lines of an Ar-ion laser; blue: 488 nm of an Ar-ion laser; red: 633 nm line of a HeNe laser). The emission above 420 nm, above 505 nm, and above 650 nm was subsequently detected in three channels.

#### 2.5.2. Flow Cytometry

The cellular uptake was examined quantitatively by flow cytometry. Briefly, Caco-2 cells were detached using a 0.05% trypsin–EDTA solution and resuspended in HBSS at a concentration of 1 × 10^6^ cells/mL. The cells were then incubated with polyplex solutions containing 5 µM siRNA for 30 min at 37 °C. Between every step the cells were washed twice with HBSS. Cellular fluorescence intensity was subsequently measured using a Guava EasyCyte 6HT-2L flow cytometer (Merck Ltd., Darmstadt, Germany). All experiments were performed independently in triplicate.

### 2.6. Investigation of the Endosomal Escape

The endosomal escape of the cargo molecules was investigated by the calcein-release assay, based on the method of Salomone et al. [29]. To simulate our previous investigations, Caco-2 cells were seeded 4 days before the experiment on 12-well plates, and were then incubated with 250 µM calcein and 100 µg/mL QABCDPS or 5 µg/mL PEI solution in HBSS for 4 h and 24 h at 37 °C. HeLa cells were seeded 24 h before the experiment on 12-well plates and were then incubated with the same solutions in Opti-MEM media for 4 h and 24 at 37 °C. After the incubation time the cells were washed with PBS and trypsinized with 0.05% trypsin–EDTA solution. The samples were then washed once with HBSS; propidium iodide (PI) at a final concentration of 1 µg/mL was added to the samples and the cellular fluorescence was analyzed by Guava Easy Cyte 6HT-2L flow cytometer (Merck Ltd., Darmstadt, Germany) in three independent experiments. Mean fluorescence intensities (MFIs) of intracellular calcein and the percent of PI-positive and -negative cells were quantified on a green (calcein) vs. red (PI) fluorescence dot plot. The results revealed distinct subpopulations:

(i) Calcein-high/PI-negative cells indicative of endosomal escape and cytosolic dye distribution;
(ii) Calcein-low/PI-negative cells representing stressed or partially membrane-compromised cells;
(iii) PI-positive dead cells with low calcein signal.

### 2.7. Investigation of the GAPDH Expression

In this experiment 80,000 HeLa cells/well were seeded on round glass cover-slips placed into 24-well plates. Four days later, cells were treated with 50 nM GAPDH siRNA in Opti-MEM media or polyplexes formulated with Lipofectamine™ RNAiMAX Transfection Reagent or QABCDPS and PEI for 48 h at 37 °C. After treatment, cells were fixed in methanol: acetone 50%:50% for 5 min at −20 °C. The non-specific binding places were blocked with fetal bovine serum for 15 min at room temperature. After blocking, cells were incubated in the 0.5 µg/mL anti-GAPDH primary antibody (rabbit polyclonal IgG, Sigma-Aldrich, Budapest, Hungary) for 1 h at 37 °C and then with the 5 µg/mL secondary antibody (Alexa Fluor 488 goat anti- rabbit polyclonal IgG, Thermo-Fisher Scientific, Waltham, MA, USA) for 1 h at 37 °C. The cell nuclei were stained with 283 nM DAPI for 10 min at room temperature. Between every step the cells were washed with HBSS. For the final step, the round glass cover-slips were glued to glass microscope slides. Fluorescence microscopic measurements were carried out by Zeiss AxioScope A1 (Carl Zeiss Microscopy GmbH, Jena, Germany) fluorescent microscope. The following filters were used to examine the samples: DAPI: excitation G 365 nm, emission BP 445/50 nm; fluorescein: excitation BP 470/40 nm, emission BP525/50 nm.

### 2.8. Investigation of the Green Fluorescent Protein (GFP) Expression

#### 2.8.1. Fluorescence Microscopy

In this experiment 40,000 HeLa cells/well were seeded on round glass cover-slips placed into 24-well plates. When the cells reached the appropriate confluence, they were treated with 2 µg/mL mRNA in Opti-MEM medium with polyplexes formulated using Lipofectamine™ MessengerMAX™ Transfection Reagent or the polymers at different NP for 48 h at 37 °C. After the incubation time, cells were fixed with 3.7% paraformaldehyde solution for 15 min at room temperature and cell nuclei were stained with 283 nM DAPI for 10 min at room temperature. Between the steps the cells were washed three times with HBSS. For the last step, the round glass cover-slips were mounted on glass microscope slides. Fluorescence microscopic measurements were carried out by Zeiss AxioScope A1 (Carl Zeiss Microscopy GmbH, Jena, Germany) fluorescent microscope. The following filters were used to examine the samples: DAPI: excitation G 365 nm, emission BP 445/50 nm; fluorescein: excitation BP 470/40 nm, emission BP525/50 nm.

#### 2.8.2. Flow Cytometry

In this experiment 100,000 HeLa cells/well were seeded on 12-well plates. After four days, when the cells reached the appropriate confluence, they were treated with 2 µg/mL mRNA in Opti-MEM media or polyplexes formulated with Lipofectamine™ MessengerMAX™ Transfection Reagent or polymers at different NP for 48 h at 37 °C. After the incubation period, the cells were washed twice with phosphate buffered saline (PBS) and trypsinized with 0.05% trypsin–EDTA solution. The samples were then washed once with HBSS and the cellular fluorescence was analyzed by Guava Easy Cyte 6HT-2L flow cytometer (Merck Ltd., Darmstadt, Germany) in three independent experiments. Viable cells were gated based on the forward scatter (FSC) vs. side scatter (SSC) dot plot and used for GFP expression analysis. Fluorescence intensity of the samples was normalized to that of viable untreated control cells.

### 2.9. Statistical Analysis

For statistical analysis GraphPad Prism 9 software (GraphPad Software Inc., San Diego, CA, USA) was used. Data are presented as means ± SD. Comparison of the groups was performed by using one-way ANOVA and Dunnett’s multiple comparison *post hoc* test. Differences were considered significant at *p* < 0.05. * *p* < 0.05; ** *p* < 0.01; *** *p* < 0.001; **** *p* < 0.0001.

## 3. Results

### 3.1. Cytotoxicity

**Figure 1:**
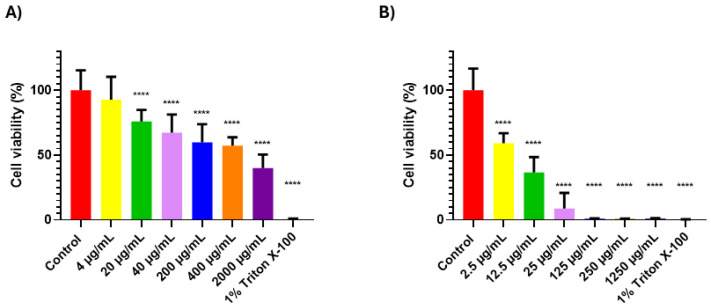
Cytotoxicity of QABCDPS (**A**) and PEI (**B**) on HeLa cervical epithelial cells after long-term, 48 h treatment, measured by the MTT method. Values are expressed as means ± SD (n = 3). **** *p* < 0.0001.

The cytotoxicity of the applied polymers was investigated using the MTT method on HeLa cervical epithelial cells. During the tests, we examined the long-term, 48 h effects of polymer solutions in different concentrations on cell viability. For QABCDPS (Figure 1A), the 20 µg/mL concentration significantly reduced cell viability, but it only decreased below 70% at higher concentrations. For PEI (Figure 1B), even the concentration of 2.5 µg/mL was significantly cytotoxic. The IC50 value of the PEI polymer is 7.6 µg/mL while for the QABCDPS is 32.4 µg/mL.

### 3.2. Properties of the Polyplexes

**Figure 2:**
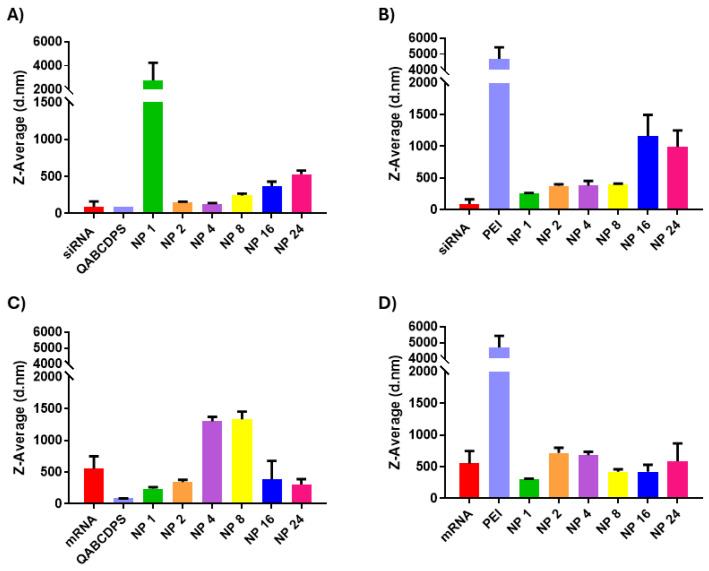
Size distribution of the formulated polyplexes, measured by DLS method. (**A**) QABCDPS-siRNA, (**B**) PEI-siRNA, (**C**) QABCDPS-mRNA, (**D**) PEI-mRNA.

The formulated polyplexes were characterized by their size distribution and zeta potential. Based on the size distribution measurements, in the case of siRNA the size of the formulated polyplexes increased with the addition of polymer, but fluctuations could be observed. Interestingly, in the case of QABCDPS, the NP 1 polyplex showed an unexpectedly large hydrodynamic diameter, which decreased drastically from NP 2, showing the condensation of the molecules and started to increase again at NP 8. PEI also had an unusual size at the applied 100 µM concentration, probably due to its association and large hydrate shell (Figure 2A,B). It can also be observed that the size of the mRNA and the polyplexes continuously increased with the addition of the polymers, and then suddenly decreased at NP 16 and NP 8 for QABCDPS and PEI, respectively. Presumably, up to this ratio, the added polymers bound to the mRNA, and then, at NP 16 and NP 8 completely surrounded and condensed it, which caused a decrease in the hydrodynamic diameter of the mRNA molecule and the polyplex (Figure 2C,D; see Supplementary Table S1 for additional data).

**Figure 3:**
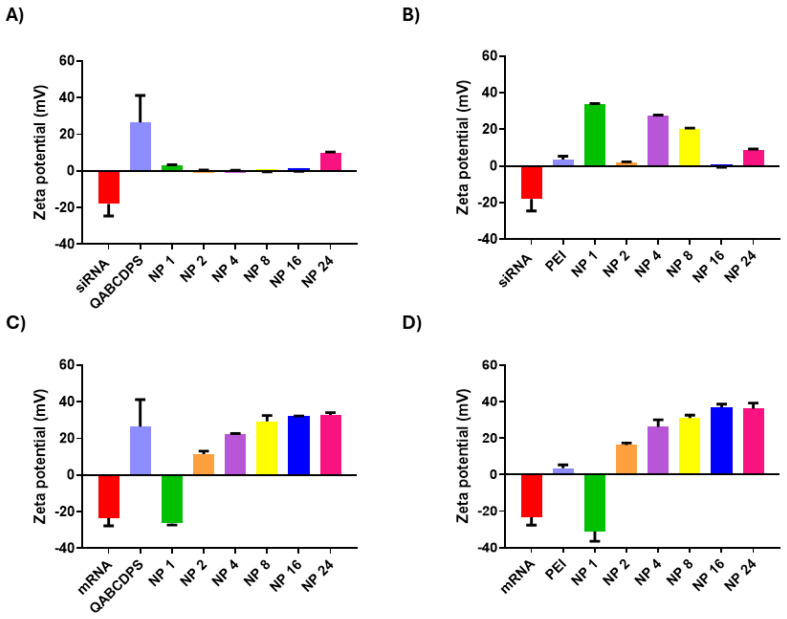
Zeta potential of the formulated polyplexes. (**A**) QABCDPS-siRNA, (**B**) PEI-siRNA, (**C**) QABCDPS-mRNA, (**D**) PEI-mRNA.

Based on the zeta potential measurements, RNA molecules exhibited a negative zeta potential, while polymers exhibited a positive zeta potential. QABCDPS had a larger positive zeta potential compared to PEI: 27 ± 15 mV and 3.5 ± 1.8 mV, respectively. For the polyplexes formulated with siRNA and QABCDPS, the zeta potential was around 0 mV between NP 1 and NP 16, only NP 24 showed a higher positive value (Figure 3A). For polyplexes formed with PEI and siRNA, positive zeta potentials were measured, but the values fluctuated. Interestingly, NP 1 polyplex had higher positive value than PEI, and further increase in NP caused a decreasing trend in zeta potential (Figure 3B). For mRNA with added polymers, the negative charge of the RNA molecule was changed to positive. For both polymers, the system was saturated at ratio NP 16 (for QABCDPS and PEI), since the zeta potential values did not increase above this ratio. mRNA showed much better response in zeta potential to the addition of polymers at increasing NP compared to siRNA, which may be related to the different size and structure of siRNA and mRNA (Figure 3C,D).

**Figure 4:**
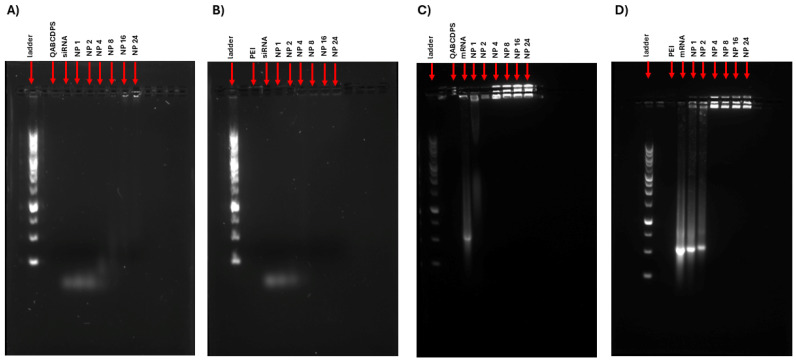
The interaction between the polymers and the RNA molecules investigated by gel electrophoresis. (**A**) QABCDPS-siRNA, (**B**) PEI-siRNA, (**C**) QABCDPS-mRNA, (**D**) PEI-mRNA.

The interaction between the RNA molecules and the polymers was investigated by gel electrophoresis. This method is a separation technique according to size in an electric field. For siRNA, the QABCDPS (Figure 4A) at ratio NP 16 and the PEI (Figure 4B) at ratio NP 4 were able to bind the siRNA molecule completely, because of its large size it could not move from the frontline. For mRNA, the QABCDPS (Figure 4C) at ratio NP 2 and PEI (Figure 4D) at ratio NP 4 were bound to the polymer molecule.

### 3.3. Cellular Uptake of siRNA Polyplexes and QABCDPS

**Figure 5:**
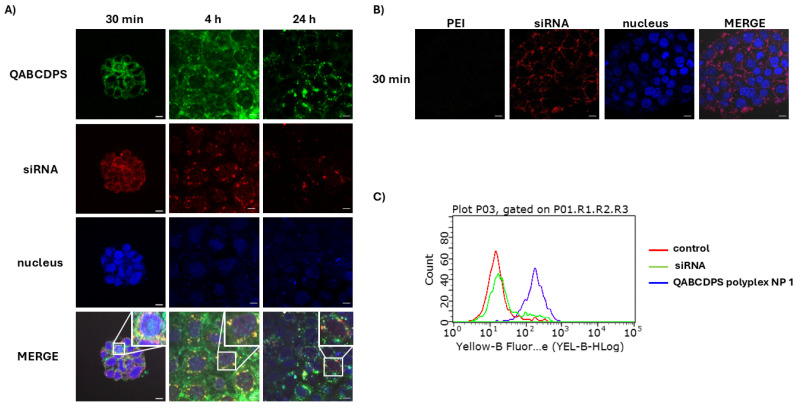
Confocal microscopic images of Caco-2 cells treated with siRNA polyplexes formulated with fluorescein-labeled QABCDPS after 30 min, 4 h, and 24 h of incubation (**A**) and PEI after 30 min of incubation (**B**). Histogram of the cellular uptake of siRNA polyplexes formulated with QABCDPS measured by flow cytometry (**C**). Blue: cell nuclei, green: QABCDPS, red: siRNA, yellow pixels show the colocalization of QABCDPS and siRNA. Inserts on panel A show the magnified image of cells containing vesicles with polyplexes. The scale bar is 10 µm.

The cellular internalization of the siRNA polyplexes was investigated by confocal microscopy. A fluorescein-labeled derivative of QABCDPS was available for these experiments; thus, its colocalization with the labeled siRNA could be investigated; however, higher concentrations for both polymers and siRNA were required to acquire a detectable fluorescent signal. The polyplexes formulated with different polymers at NP 1 (for QABCDPS and PEI) showed various cellular internalization patterns. The QABCDPS polyplexes entered the cells and localized in vesicles in the cytoplasm. From the overlap of the green and red signals, which is shown in yellow, we conclude that the polymer and siRNA were colocalized after short-term (30 min) and long-term (4 h and 24 h) incubation (Figure 5A). Using the fluorescein-labeled derivative of QABCDPS provided the opportunity to investigate the intracellular fate of the polyplex for up to 24 h. This derivative allowed the detection of the intracellular behavior of both the polymer and the polyplex during this period. Polyplexes formulated with PEI either bound to the cell membrane or formed larger aggregates in membrane ligations after 30 min of incubation (Figure 5B). The cells treated with PEI for 30 min appear shrunken and rounded, providing visual signs of acute cytotoxicity. Fluorescently labeled PEI was not available, so the intracellular fate of PEI could not be detected in this case (see Supplementary Figures S1–S3 for additional data).

The cellular uptake results were confirmed by flow cytometry. For this, the cells were treated with siRNA polyplexes for 30 min. However, to obtain a detectable fluorescence signal, a high concentration of siRNA (5 µM), and consequently a high concentration of polymer, had to be used. The cellular fluorescence intensity arising from the internalized siRNA was measured. For the QABCDPS polyplexes, the fluorescence signal was stronger as compared to the control sample. This means that these polyplexes were not removed by washing after incubation; instead, they were taken up into the cells (Figure 5C). For the PEI polyplexes, no living cells were detected in the samples because the applied 5 µM concentration of the polymer was toxic, which is in accordance with the cytotoxicity results. Unfortunately the application of lower concentrations of fluorescently labeled siRNA and PEI was not suitable for flow cytometry measurements. The uptake capacity of Caco-2 and HeLa cells was further investigated using FITC-QABCDPS and FITC-dextran in flow cytometry experiments. Interestingly, Caco-2 cells internalized significantly higher amounts of FITC-dextran, whereas HeLa cells showed higher uptake of FITC-QABCDPS compared to Caco-2 cells (Supplementary Figure S4).

### 3.4. GAPDH Expression

**Figure 6:**
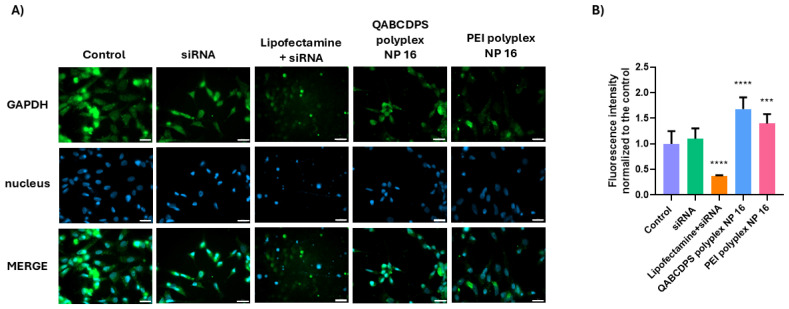
(**A**) Fluorescence microscopic images of HeLa cells treated with siRNA polyplexes for 48 h. Polyplexes formulated with QABCDPS and PEI at NP 16 were unable to influence the GAPDH expression. Blue: cell nuclei, green: GAPDH protein. (**B**) Effects of the polyplex treatment on GAPDH expression in HeLa cells. Values are expressed as means ± SD (n = 10). *** *p* < 0.001; **** *p* < 0.0001. The scale bar is 20 µm.

The intracellular effects on gene silencing of the siRNA polyplexes were investigated via the expression of GAPDH protein by fluorescence microscopy. For this, the cells were treated with polyplexes at NP 16 (for QABCDPS and PEI) for 48 h, and then the protein was labeled using an immunofluorescence method. The quantitative analysis of the images revealed that QABCDPS and PEI polyplexes were unable to decrease the expression of the protein (Figure 6).

### 3.5. GFP Expression

**Figure 7:**
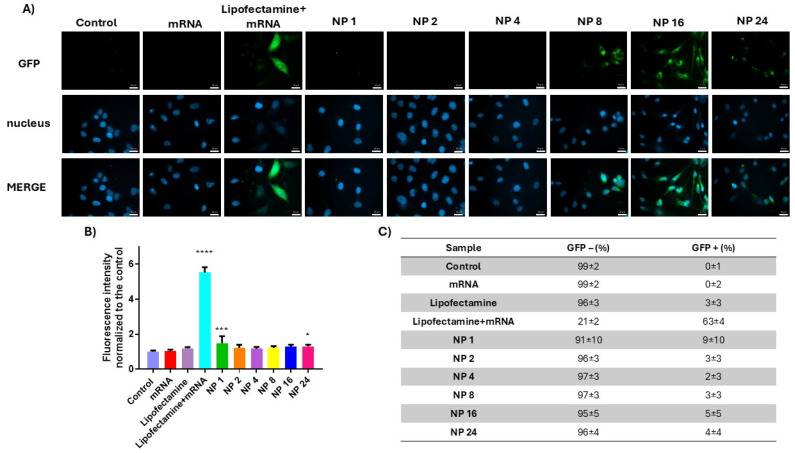
Fluorescence microscopic images of HeLa cells treated with mRNA polyplexes formulated with QABCDPS (**A**). Blue: cell nuclei, green: green fluorescent protein. Effects of the polyplexes on the GFP expression after polyplex treatment containing 2 µg mRNA, measured by flow cytometry (**B**). Values are expressed as means ± SD (n = 3). * *p* < 0.05; *** *p* < 0.001; **** *p* < 0.0001. The scale bar is 20 µm. (**C**) The average % of GFP- positive and GFP- negative cells. Values are expressed as means ± SD (n = 3).

**Figure 8:**
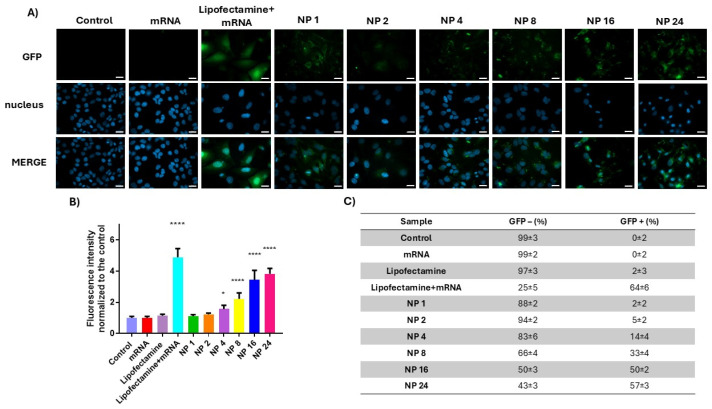
Fluorescence microscopic images of HeLa cells treated with polyplexes formulated with PEI polymer (**A**). Blue: cell nuclei, green: green fluorescent protein. Effects of the polyplexes on the GFP expression after polyplex treatment containing 2 µg mRNA, measured by flow cytometry (**B**). Values are expressed as means ± SD (n = 3). * *p* < 0.05; **** *p* < 0.0001. The scale bar is 20 µm. (**C**) The average % of GFP- positive and GFP- negative cells. Values are expressed as means ± SD (n = 3).

The intracellular effects of the mRNA polyplexes were investigated via the expression of the green fluorescent protein (GFP) by fluorescence microscopy. For this, the HeLa cells were treated with polyplexes at different NPs for 48 h. For the polyplexes formulated with QABCDPS, it can be observed that after treatment with the polyplexes at NP 8, NP 16, and NP 24, a green signal was detected in the cytoplasm; i.e., the protein was expressed (Figure 7A). For the polyplexes formulated with the PEI (Figure 8A), a green signal was already detected in the cytoplasm vesicles after the polyplex treatment at NP 1, so the green fluorescent protein was transcribed from the mRNA. With an increase in the NP, i.e., with an increase in the amount of added polymer, a change in the shape of the cells could be observed, indicating the toxic effects of the polymer on cells. This result is in accordance with the cell viability test performed with the MTT method, according to which PEI is toxic to HeLa cells in increasing concentrations.

The results were confirmed by flow cytometric measurements, where the cellular fluorescence intensity caused by the green fluorescent protein was measured. QABCDPS polyplex treatments at NP 1 and NP 24 containing 2 µg of mRNA, increased significantly the measured fluorescence intensity compared to the control, untreated cells (Figure 7B). For the polyplexes formulated with PEI, the cellular fluorescence intensity, i.e., the amount of expressed protein, increased significantly after treatment at 2 μg of mRNA, starting from an NP 4 and then upward (Figure 8B).

## 4. Discussion

With the appearance of the mRNA vaccine against the SARS-CoV-2 virus, the research into RNA-based therapies and vaccines has expanded [30]. Most of the carrier systems currently on the market or under development are liposomes or lipid-based nanoparticles. These have many side effects related to the carrier part, which can be avoided with polymer-based systems. These polymers are usually cationic molecules, just like the positively charged cyclodextrin derivatives. Due to their large molecular size and polyanionic nature, RNA molecules are difficult to transfect into cells and are also unprotected against RNases. Therefore, an efficient and safe transport system is needed. If the polyplex is taken up into the cells and reaches the lysosomes, they can show the so-called proton sponge effect. During the process, the protonable cationic polymers are protonated, resulting in the uptake of water into the lysosomes, which swell and their membrane ruptures, so that the polyplex is released into the cytoplasm [31]. Polyethylenimine is a widely used reference polymer in the field of polymer-based nucleic-acid delivery; however, it has many limitations [32]. The branched PEI contains primary, secondary, and tertiary amino groups and has surfactant-like properties. Its cellular toxicity is strongly linked to the cationic and amphiphilic properties of the polymer [33], causing the disruption of lipid bilayers [34]. It is in agreement with our findings that concentrations higher than 2.5 µg/mL PEI caused significant cellular toxicity. Unlike the PEI, the QABCDPS exhibited significantly lower cytotoxicity. However, it should be noted that in our experimental setup, the prolonged serum deprivation in the Opti-MEM medium can induce metabolic stress and suppress proliferation, potentially confounding IC_50_ estimates. This effect becomes evident after 48 h of incubation and is reflected in the MTT results. Therefore, these data should be interpreted with caution and compared carefully with literature reports obtained using complete growth medium. MTT results are in accordance with our earlier findings, where quaternary amino-β-cyclodextrin monomer (QABCD) was tested on Caco-2 cells and its IC50 value was higher than 200 mM [35]. The investigated QABCDPS, a polymeric form of QABCD monomer units containing identical quaternary ammonium groups, proved to be a logical and promising alternative for RNA delivery. QABCDPS is not protonable but permanently charged due to the quaternary ammonium groups, while the protonation of PEI is heavily pH-dependent. The amines of PEI are only partially protonated at neutral pH.

During our research work, we aimed to investigate the siRNA and mRNA carrying capacity of QABCDPS and to compare it with PEI, which is a widely used carrier. Despite the fact that QABCDPS confers a simple formulation opportunity to RNA delivery, its application is sparingly studied. A recent publication systematically revealed the siRNA delivery application of β-cyclodextrin monomer, dimer, and polymer derivatives functionalized with quaternary ammonium groups [26], while other studies presented its application for nasal delivery and for co-delivery of 5-fluorouracil and interleukin-2 [36]. Firstly, we formulated polyplexes with different NP, which were characterized based on their size distribution and zeta potential. These measurements were obtained in RNase-free water, because high protein content, intrinsic absorbance, and coloration of the cell culture media would have interfered with light-scattering detection. Therefore, the reported size and zeta potential values may not represent the state of polyplexes during cellular uptake, limiting direct correlation between physicochemical properties and biological outcomes. For siRNA, the size measurements showed unusual behavior of the polyplexes. QABCDPS NP 1 polyplex showed an unexpectedly large hydrodynamic diameter, which can be explained by the loose structure of the associated molecules and large hydration shell around the associations. Above this NP, the size decreased significantly, probably due to the condensation, and showed an increasing trend up to NP 24. The hydrodynamic diameter of the PEI-siRNA polyplexes increased with increasing NP up to NP 16. These measurements were obtained with high siRNA concentrations, under non-physiological conditions that may cause aggregation and should be interpreted only qualitatively. For mRNA, QABCDPS polyplexes showed an increase in size up to NP 8 and then decreased at NP 16 (Figure 2C), while PEI polyplexes showed a uniform size distribution.

From the zeta potential measurements, it can be concluded that, at first, QABCDPS had a much more positive zeta potential, compared to PEI. It is caused by the permanent positive charges of QABCDPS. QABCDPS neutralized the negative zeta potential of siRNA, while the addition of PEI to siRNA caused an increase in zeta potential. It can be caused by the association of numerous PEI molecules to the siRNA, losing their hydrate shell, but at higher NP, larger and more condensed associates with less surface charge were formed, especially at NP 16 and NP 24 (Figure 2B and Figure 3B). The negative charge of the mRNA molecules became positive with the addition of the positively charged polymers, and this value also gradually increased (Figure 3). Based on the obtained results, we assume that the mRNA system was saturated for both QABCDPS and PEI at NP 16 (for QABCDPS and PEI); at this ratio the polymers completely covered the RNA and condensed it. The interaction between the polymer and the mRNA was also investigated using gel electrophoresis, which is a size-based separation method in an electric field. According to the results, for the QABCDPS, the polyplex with siRNA at NP 16 and mRNA at NP 2, and the polyplex with both RNA molecules at NP 4 for the PEI, completely bound the RNA molecule; it could not move farther from the front line, due to its large molecular size (Figure 4).

After the characterization of the formulated polyplexes, the cellular internalization was investigated. The polyplexes formulated with different polymers have shown different internalization. The polyplexes with QABCDPS entered the cells and localized in vesicles after a short- and long-term treatment (Figure 5). In contrast, the polyplexes formulated with PEI were bound to the cell membrane and located in vesicles in the cytoplasm (also see Supplementary Data). Furthermore, after PEI-siRNA polyplex treatment, a shrunken and rounded shape appeared, which may indicate an acute cytotoxicity. Interestingly, the fluorescence signal of both QABCDPS and the siRNA could be detected after 24 h in vesicles in the cytoplasm. The siRNA still localized mainly in vesicles and colocalized partially with the QABCDPS. It indicates a strong interaction between siRNA and QABCDPS even in the cellular environment, which may decrease its escape and the ratio of free and effective siRNA. Interestingly, no siRNA uptake was detected with the quaternary amino cyclodextrin polymer in A549luc cells in an earlier study [26]. It should be mentioned that the two polymers applied in the two studies are not identical, even if the synthesis method was the same and quaternary ammonium groups were introduced by post-polymerization modification. There can be a difference between the size distribution of the polymers, as the molecular weight cutoff (MWCO) of the dialysis membranes used for purification was different (2 kDa and 14 kDa in the earlier and present study, respectively) and the DS of the quaternary amino groups was also different (4.0 and 1.5–2.0 in the earlier and present study, respectively). Differences in size distribution and substitution can significantly influence its behavior and cellular uptake.

The intracellular effects of the siRNA polyplexes was investigated via the expression of the GAPDH protein. After 48 h polyplex treatment, the polyplexes were unable to decrease the protein expression compared to the untreated control (Figure 6).

The mRNA polyplex intracellular effects were investigated through the expression of the green fluorescent protein. After treatment with polyplexes formulated with QABCDPS, the protein was translated and expressed from the NP 8 (Figure 7), while after formulation with PEI, the protein was expressed after treatment with the NP 1 (Figure 8). Interestingly, the GFP expression induced by QABCDPS polyplexes corresponds to the size of the polyplexes. Polyplexes at NP 1 and N 24 caused a significant increase in GFP expression and had the smallest hydrodynamic diameter (Figure 2C and Figure 7B).

Overall, we successfully formulated simple cyclodextrin polymer-based RNA delivery systems, which carry the RNA molecules and deliver them into the cytoplasm. Simplicity and safety of polyplexes could be their main advantages, as they require only two components and a simple formulation process. However, it should be noted that the investigated systems have both advantages and disadvantages. The greatest advantage of the QABCDPS-based polyplexes is that they are less toxic than the gold-standard PEI. QABCDPS binds both siRNA and mRNA, but the behavior of mRNA polyplexes was more consistent. It can be explained by their different structure and the presence of the permanent charge of quaternary ammonium groups in QABCDPS. The larger size of QABCDPS also contributes to the optimal interaction of the polymer with mRNA. It was also reflected in the protein expression levels caused by the polyplexes, which were lower for the QABCDPS compared to the PEI, probably due to the lower free intracellular level of mRNA. A key limitation in this study lies in the use of different cell lines across experimental setups. Previously, we reported that Caco-2 cells showed significantly higher FITC-dextrane uptake and a pronounced lysosomal accumulation, when compared to HeLa cells. Based on these previous results, we performed the uptake studies on Caco-2 cells; therefore, HeLa cells were chosen for the functional assays. Additionally, DLS measurements required a high siRNA concentration, which may have induced aggregation and affected particle size distribution, representing a potential source of bias. Furthermore, PEI induced cellular stress, evidenced by morphological changes and calcein-release assay results, which may have affected GFP expression and represents another limitation in the study. This study compared the applicability of QABCDPS for siRNA and mRNA delivery for the first time and the limitations were assessed. Based on these results and by the help of a rational polymer design, new and more efficient polymers can be synthesized with a suitable safety profile and carrier properties.

## Data Availability

The original contributions presented in this study are included in the article/Supplementary Material. Further inquiries can be directed to the corresponding author.

## Supplementary Materials

The following supporting information can be downloaded at https://www.mdpi.com/article/10.3390/pharmaceutics18060713/s1, Figure S1: QABCDPS polyplex 30 min; Figure S2: QABCDPS polyplex 24 H; Figure S3: PEI polyplex 30 min; Figure S4: FITC-dextrane and QABCDPS endocytosis; Figure S5: Endosomal escape; Figure S6: QABCDPS polyplex histograms; Figure S7: PEI polyplex histograms; Table S1: Comprehensive summary table presenting key physicochemical properties (hydrodinamic diameter, PDI, zeta potential and gelelectrophoresis) and biological activity of the formulated polyplexes; Table S2: Quantitative analysis of calcein/PI-defined cell subpopulations (%).

## Abbreviations

The following abbreviations are used in this manuscript:

siRNA	Small Interfering Ribonucleic Acid
mRNA	Messenger Ribonucleic Acid
QABCDPS	Quaternary ammonium β-cyclodextrin polymer
PEI	Polyethylenimine
PA-polymer	β-cyclodextrin polymer functionalized with primary amine groups
GAPDH	Glyceraldehyde 3-phosphate dehydrogenase
GFP	Green fluorescent protein
Caco-2	Caco-2 intestinal epithelial cell line
HeLa	HeLa human cervix epithelioid carcinoma cell
DMEM	Dulbecco’s modified Eagle’s medium
MTT assay	3-(4,5-dimethylthiazolyl-2)-2,5-diphenyltetrazolium bromide assay
NP	Nitrogen/phosphate ratio
DLS	Dynamic light scattering
ZP	Zeta potential
TAE buffer	Tris base, acetic acid and ethylenediaminetetraacetic acid buffer
HBSS	Hank’s Balanced Salt Solution
DAPI	4′,6-diamidino-2-phenylindole
