# Supplementary material for: Comparison of the siRNA and mRNA Carrying Capacity of Quaternary Ammonium β-Cyclodextrin Polymer and Polyethylenimine
Source: Pharmaceutics. 2026 Jun 10;18(6):713. doi: 10.3390/pharmaceutics18060713 (PMC13307255; doi:10.3390/pharmaceutics18060713)
Supplement: Supplementary file 1 [file pharmaceutics-18-00713-s001.zip › Supplementary Table S1.pdf]

Supplementary Table S1: Comprehensive summary table presenting key physicochemical properties (hydrodinamic diameter, PDI, zeta potential and gelelectrophoresis) and biological activity of the formulated polyplexes.

	Hydrodinamic diameter (nm)	PDI	Zeta potential (mV)	Gelelectrophoresis (+ retarded the RNA/- not retarted the RNA)	Biological activity (+ effective/- ineffective)
**siRNA**	89±74	0.9±0.2	-18±7	not relevant	-
**QABCDPS**	86±0.9	0.4±0.006	27±15	-	not relevant
**NP 1**	2772±1464	0.8±0.2	3.2±0.2	-	not relevant
**NP 2**	152±8	0.4±0.04	0.4±0.09	-	not relevant
**NP 4**	133±10	0.6±0.04	0.3±0.1	-	not relevant
**NP 8**	243±25	0.5±0.09	-0.3±0.2	-	not relevant
**NP 16**	371±60	0.6±0.2	-0.1±0.07	+	-
**NP 24**	530±49	0.7±0.04	10±0.3	+	not relevant
**PEI**	4694±732	0.4±0.05	3.5±1.8	-	not relevant
**NP 1**	260±4	0.5±0.004	33.6±0.5	-	not relevant
**NP 2**	376±25	0.5±0.1	2.1±0.2	-	not relevant
**NP 4**	380±74	0.5±0.1	28±0.3	+	not relevant
**NP 8**	399±12	0.4±0.01	20.2±0.6	+	not relevant
**NP 16**	1156±337	0.9±0.1	-0.4±0.2	+	-
**NP 24**	989±260	0.9±0.07	8.7±0.6	+	not relevant

	Hydrodinamic diameter (nm)	PDI	Zeta potential (mV)	Gelelectrophoresis (+ retarded the RNA/- not retarted the RNA)	Biological activity (+ effective/- ineffective)
**mRNA**	552±197	0.7±0.2	-23±4	not relevant	-
**QABCDPS**	86±0.9	0.4±0.006	27±15	-	not relevant
**NP 1**	238±25	0.3±0.01	-26±0.9	-	+
**NP 2**	347±32	0.4±0.03	11±2	+	-
**NP 4**	1301±69	0.8±0.04	22±0.5	+	-
**NP 8**	1329±124	0.9±0.07	29±3	+	-
**NP 16**	387±291	0.6±0.08	32±0.2	+	-
**NP 24**	306±84	0.6±0.04	33±1	+	+
**PEI**	4694±732	0.4±0.05	3.5±1.8	-	not relevant
**NP 1**	298±13	0.4±0.01	-31±5	-	-
**NP 2**	720±78	0.7±0.04	16±1	-	-
**NP 4**	683±53	0.6±0.04	26±4	+	+
**NP 8**	423±38	0.5±0.01	31±2	+	+
**NP 16**	423±108	0.4±0.02	37±2	+	+
**NP 24**	587±281	0.6±0.2	36±3	+	+
