# Supplementary material for: Comparison of the siRNA and mRNA Carrying Capacity of Quaternary Ammonium β-Cyclodextrin Polymer and Polyethylenimine
Source: Pharmaceutics. 2026 Jun 10;18(6):713. doi: 10.3390/pharmaceutics18060713 (PMC13307255; doi:10.3390/pharmaceutics18060713)
Supplement: Supplementary file 1 [file pharmaceutics-18-00713-s001.zip › Supplementary Table S2..pdf]

Supplementary Table S2.

Quantitative analysis of calcein/PI-defined cell subpopulations (%)

	%PI-negative cells	%PI-positive cells	% PI-negative, calcein low (stressed) cells
**HeLa 24 h control**	82.2±1.4	6.6±0.8	4.9±0.3
**HeLa 24 h calcein**	89.1±0.7	5.8±0.3	2.4±0.3
**HeLa 24h QABCDPS**	24.6±2.2	16.0±0.2	49.9±1.9
**HeLa 24h PEI**	4.7±0.4	23.9±0.4	67.1±1.2
**HeLa 4 h control**	81.7±2.2	5.1±0.1	4.7±0.6
**HeLa 4 h calcein**	89.6±1.3	3.6±0.7	3.5±0.6
**HeLa 4h QABCDPS**	48.1±0.9	4.6±0.6	34.1±0.8
**HeLa 4h PEI**	5.0±0.8	6.6±0.7	82.8±1.9
**Caco-2 24h control**	3.2±5.6	62.1±0.5	13.8±0.2
**Caco-2 24 h calcein**	47.0±5.0	15.5±2.7	13.9±0.7
**Caco-2 24 h QABCDPS**	54.2±1.6	20.5±2.6	11.6±2.7
**Caco-2 24h PEI**	34.5±2.2	20.2±0.4	33.2±2.4
**Caco-2 4h control**	74.4±1.8	6.3±1.4	6.9±1.2
**Caco-2 4 h calcein**	52.5±3.0	8.4±2.3	9,.3±3.6
**Caco-2 4 h QABCDPS**	43.9±5.5	8.3±1.1	30.6±5.2
**Caco-2 4h PEI**	46.6±6.2	9.8±0.9	34.5±4.5

The mean fluorescence intensity (MFI) of PI-negative cells was used to evaluate endosomal escape.
