# Supplementary figures and images for: Comparison of the siRNA and mRNA Carrying Capacity of Quaternary Ammonium β-Cyclodextrin Polymer and Polyethylenimine
Source: Pharmaceutics. 2026 Jun 10;18(6):713. doi: 10.3390/pharmaceutics18060713 (PMC13307255; doi:10.3390/pharmaceutics18060713)

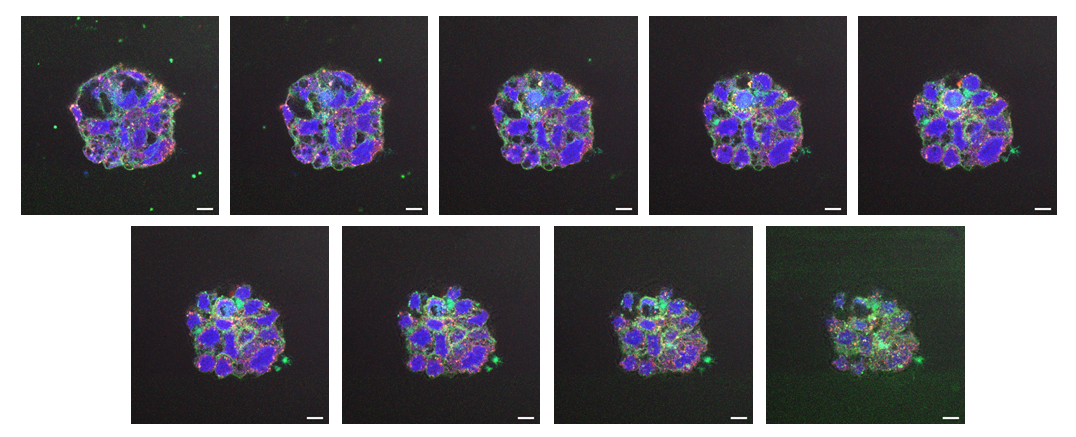

Supplement: Supplementary file 1 [file pharmaceutics-18-00713-s001.zip › Supplementary Figure S1._QABCDPS polyplex 30 min.tif]

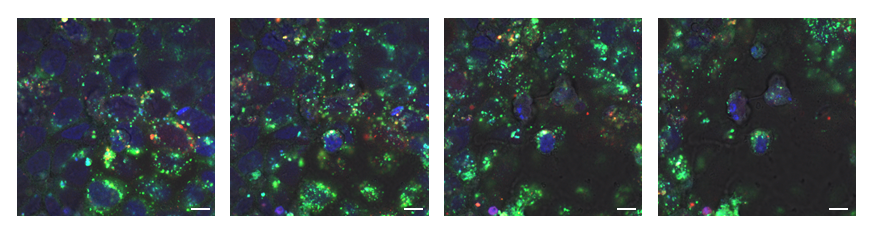

Supplement: Supplementary file 1 [file pharmaceutics-18-00713-s001.zip › Supplementary Figure S2._QABCDPS polyplex 24 h.tif]

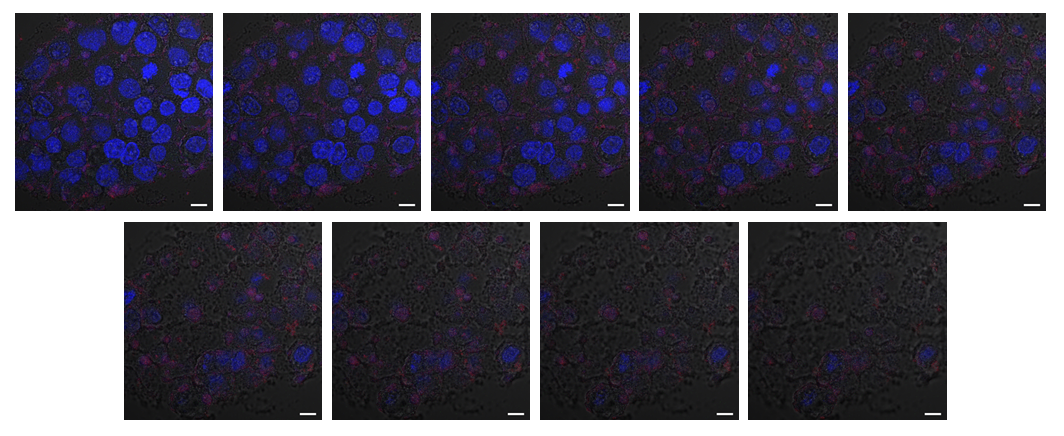

Supplement: Supplementary file 1 [file pharmaceutics-18-00713-s001.zip › Supplementary Figure S3._PEI polyplex 30 min.tif]

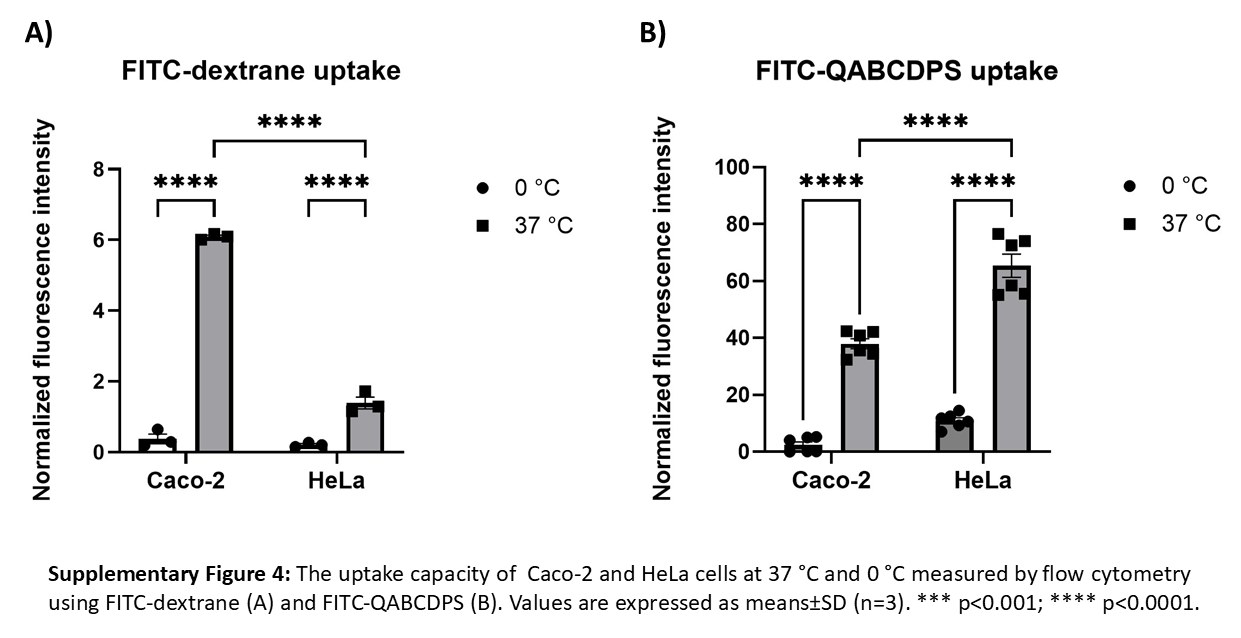

Supplement: Supplementary file 1 [file pharmaceutics-18-00713-s001.zip › Supplementary Figure S4._FITC-dextrane and QABCDPS endocytosis.tif]

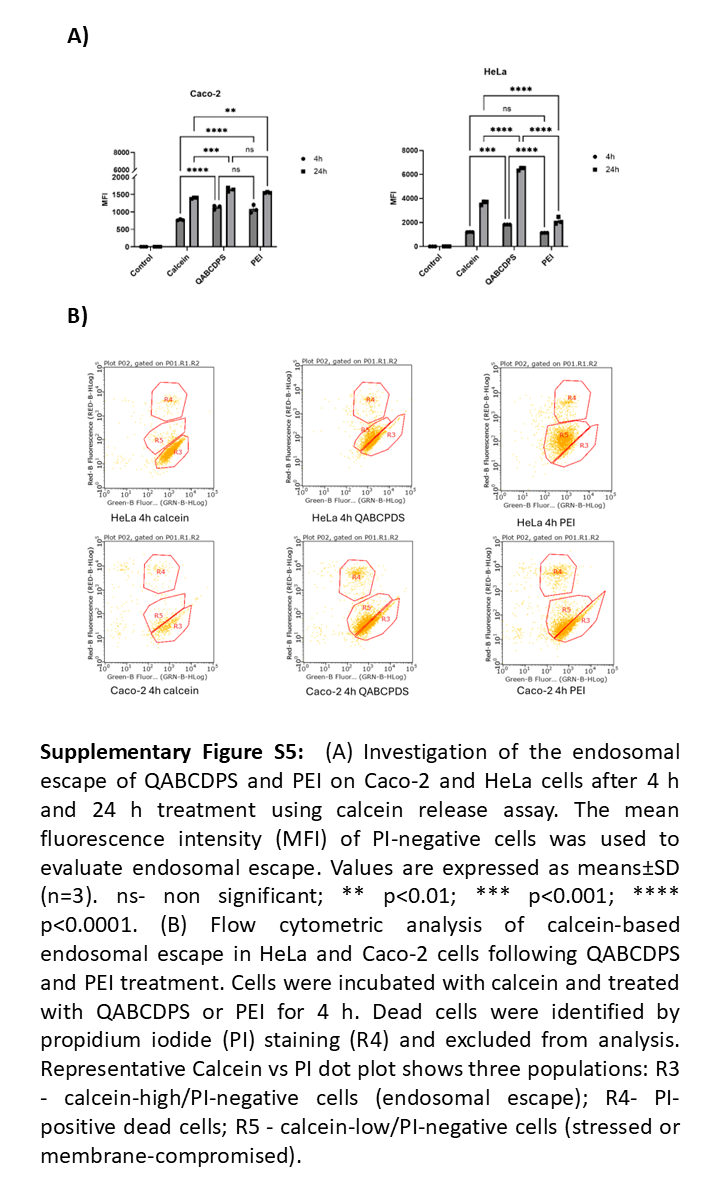

Supplement: Supplementary file 1 [file pharmaceutics-18-00713-s001.zip › Supplementary Figure S5._Endosomal escape.tif]

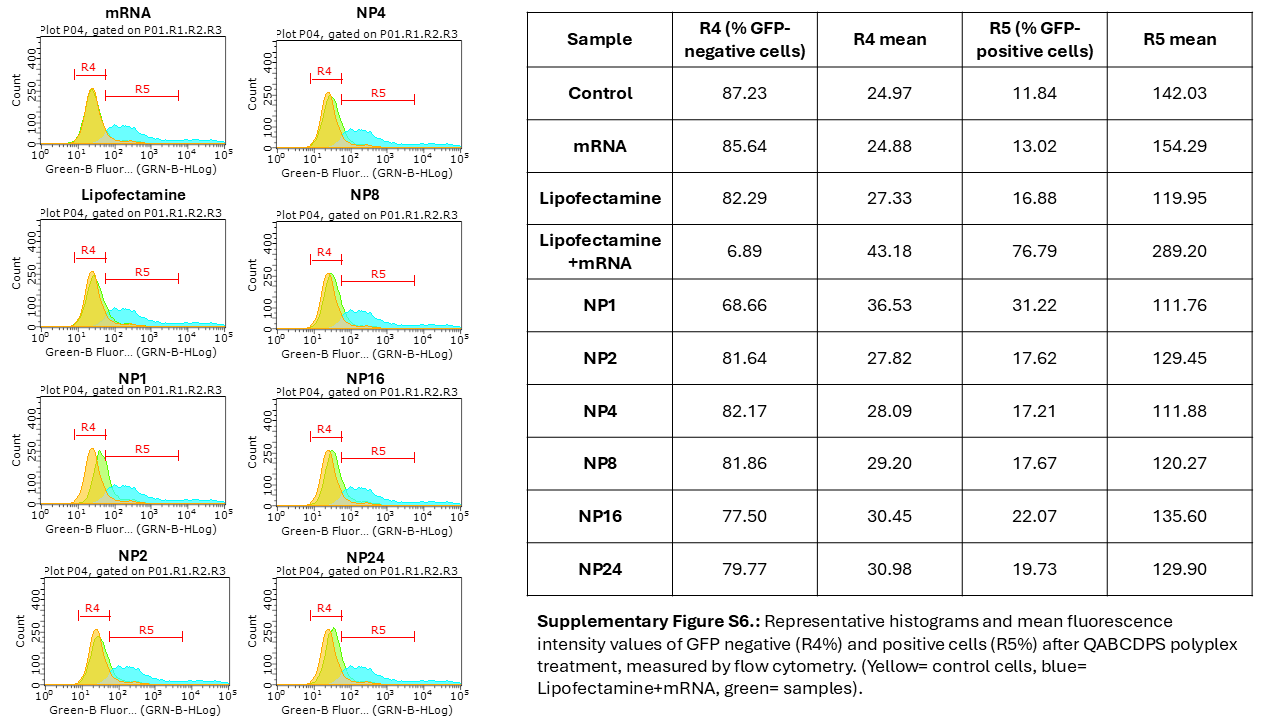

Supplement: Supplementary file 1 [file pharmaceutics-18-00713-s001.zip › Supplementary Figure S6._QABCDPS polyplex histograms.tif]

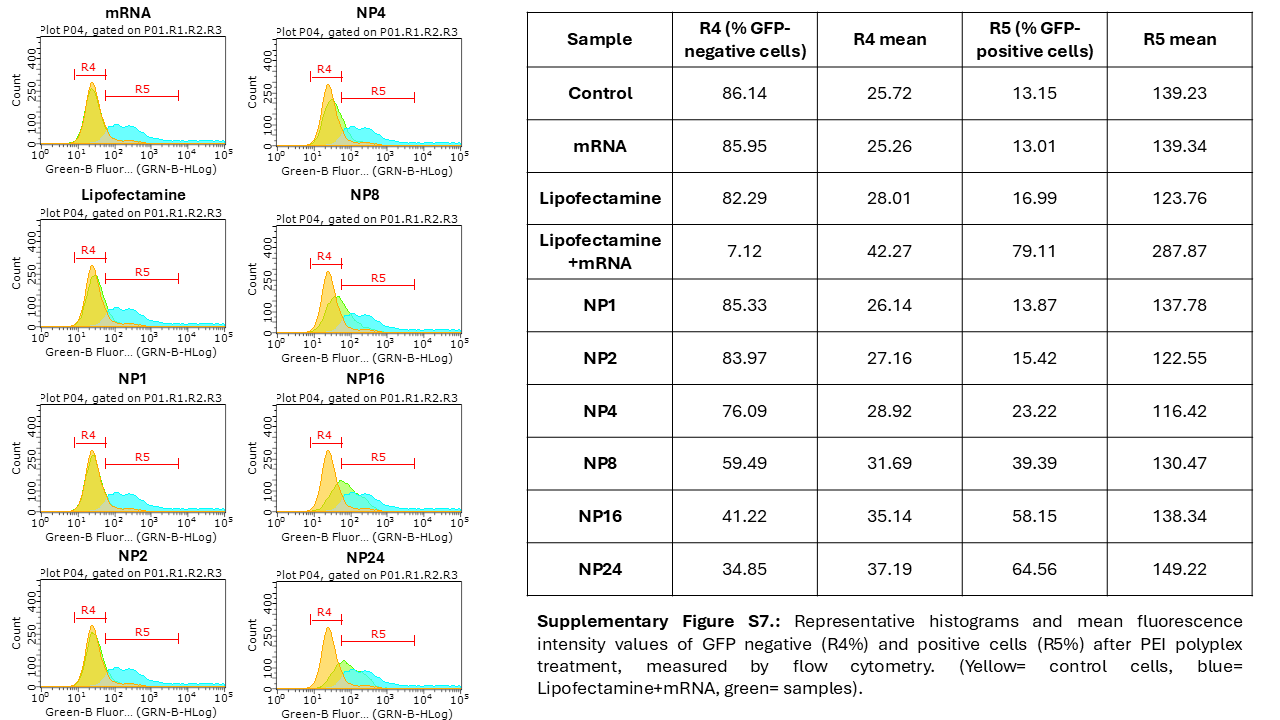

Supplement: Supplementary file 1 [file pharmaceutics-18-00713-s001.zip › Supplementary Figure S7._PEI polyplex histograms.tif]
